# Seven-year exclusivity and beyond for drugs of rare diseases in China

**DOI:** 10.3389/fphar.2023.1223056

**Published:** 2023-10-12

**Authors:** Yale Jiang, Huiyao Huang, Guo Zhao, Peiwen Ma, Liang Zhang, Shuhang Wang, Ning Li

**Affiliations:** ^1^ Clinical Trial Center, National Cancer Center/National Clinical Research Center for Cancer/Cancer Hospital, Chinese Academy of Medical Sciences and Peking Union Medical College, Beijing, China; ^2^ Zhejiang University, Hangzhou, Zhejiang, China

**Keywords:** rare disesases, policy, drug policies, China, drug marketing application

## Introduction

**FIGURE 1:**
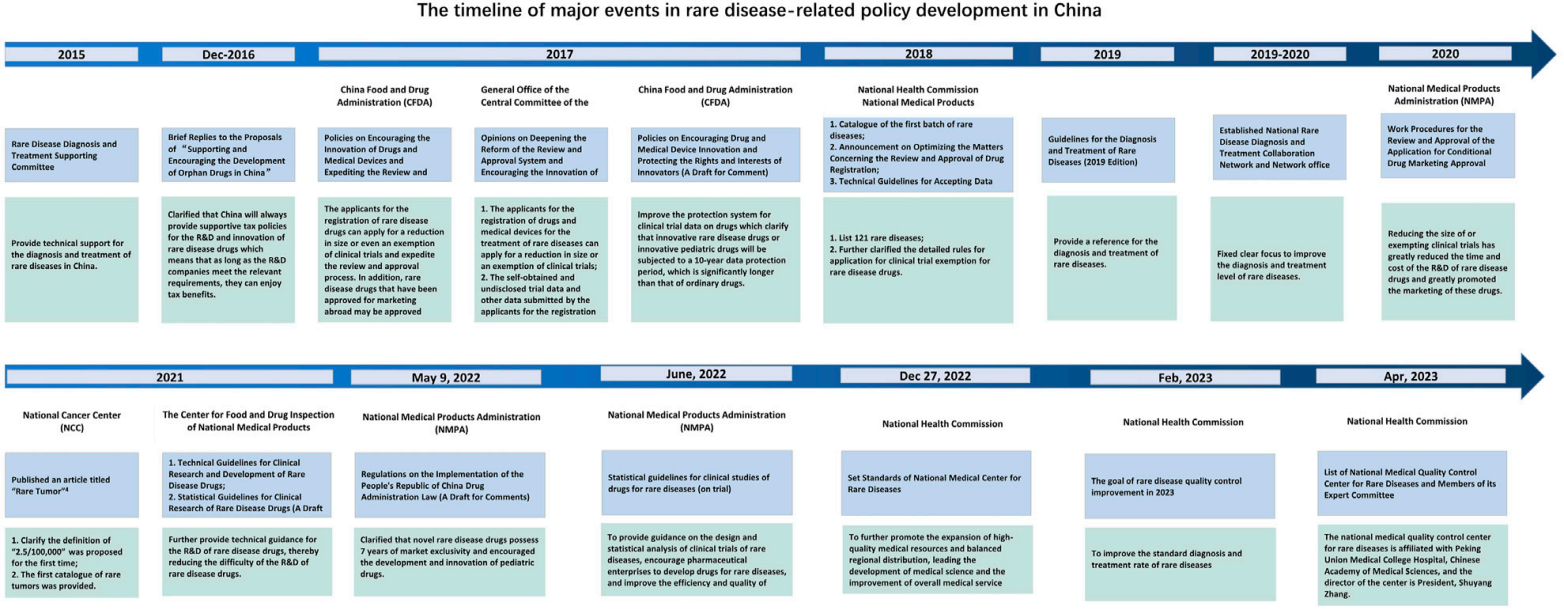
Timeline of major events in rare disease in China.

Different countries and regions have various definitions for rare diseases and rare tumors (Table 1). In 2018, the National Health Commission of China published the “Catalog for the First Batch of Rare Diseases” (National Health Commission of the People’s Republic of China, 2018). Since then, a series of policies have been successively issued (Figure 1). For example, after the implementation of the new “Drug Administration Law” on 1 December 2019, the National Medical Products Administration (NMPA) formulated the new versions of “Drug Registration Management Measures” and “Priority Review and Approval Procedures for Drug Marketing Authorization (Trial Version).” According to these documents, for rare disease drugs eligible for priority review and approval, the review and approval of marketing authorization applications will be finished within 130 days. The procedure will even be shortened to 70 days for urgently needed rare disease drugs that have been previously approved abroad. All these policies further improved the review procedure for rare disease drugs (National Medical Products Administration, 2020). In addition, the inclusion of rare disease drugs will be prioritized in the list adjustment of medicines for national basic medical insurance. This, combined with the opening of a “special channel” in Bo’ao, Hainan, improved the availability of rare disease drugs and promoted further R&D. So far, more than 60 rare disease drugs have been approved for marketing in China, of which more than 40 drugs targeting 25 rare diseases have been included in the list of medicines for national basic medical insurance.

**TABLE 1:** Definition of rare disease worldwide.

Country/Area	Type	Definition
WHO	Rare Disease	<0.65‰–1‰ of general population
CHINA	Rare Disease	121 Rare Disease listed in the “Catalogue of the first batch of rare diseases"
US	Rare Disease	<200,000
EU	Rare Disease	<5/10,000, life threatening or causes long-term suffering
JAPAN	Rare Disease	<50,000, or <1/2,500
AUSTRAI	Rare Disease	<2,000
KOREA	Rare Disease	<20,000
CANADA	Rare Disease	<1/2000

To standardize and promote drug development of rare diseases, China has promulgated a series of supportive policies. The Center for Drug Evaluation (CDE) of NMPA released the “Technical guidelines for clinical development of drugs for rare diseases” on 31 December 2021, aiming to standardize and accelerate drug development and clinical use for rare disease. Besides, to encourage drug development for rare diseases and to guide sponsors to improve R&D from the perspective of clinical study methodology, the CDE organized and developed the “Guidelines for Clinical Study Statistics of Drugs for Rare Diseases (Trial Version)”. The National Health Insurance Administration (NHA) has made many efforts to improve the affordability of drugs for rare diseases. Up to now, 56 drugs for 27 rare diseases have been included in the NHA catalogue. Nearly 40% of these rare disease drugs were included in the coverage of the basic system (basic medical insurance, supplementary medical insurance or medical assistance) since 2018 (Supplementary Table S1).

On 9 May 2022, the NPMA issued the “Regulations on the Implementation of Chinese Drug Administration Law (A Draft for Comments)” (hereinafter referred to as the Draft for Comments) (National Medical Products Administration, 2022). The regulations state that China encourages the development and innovation of rare disease drugs, supports drug marketing authorization holders to develop drugs for rare diseases, encourages the development of rare diseases into new indications for drugs that have already been marketed, and prioritizes the review and approval of rare disease drugs that are urgently needed clinically. Furthermore, during drug development and registration application, the communication between regulatory authorities and applicants will be strengthened to accelerate the marketing of rare disease drugs to meet the medication needs of this population. Most importantly, for new rare disease drugs approved for marketing, under the premise that the drug marketing authorization holder promises to ensure the supply of the drug, a market exclusivity period of up to 7 years will be given to the drug, during which the same type of drugs will not be approved for marketing. Exclusivity refers to the delaying or prohibiting of the marketing of competitive drugs during the period of drug marketing protection according to the regulations, which is an administrative protection provided by drug regulatory authorities (Center for Drug Evaluation and Research, 2022). Additionally, the NMPA has built a special channel for the review and approval of new drugs with urgent clinical needs that are approved for marketing outside of China. As of July 2022, 20 rare disease drugs previously marketed outside of China have been approved for marketing in China using data from overseas studies.

Children are worthy of attention considering rare diseases, especially rare tumors. China has 250 million children, accounting for 17.95% of the total population. The shortage of medicines for children remains a major problem in China, such as drug varieties, dosage forms, specifications, and clinical data. The Draft for Comments also encourages the R&D of pediatric drugs. First, priority will be given to the review and approval of new varieties, new dosage forms, and new specifications of medicines for children. At the same time, applicants and regulatory authorities are encouraged to communicate during drug development and registration application to accelerate the marketing of pediatric drugs and thereby meet the medication needs of pediatric patients. In terms of market protection, a market exclusivity period will be provided for innovative products. Specifically, for the first approved new variety, dosage form and specification of pediatric drug, or the drug that increases indications, uses or dosages in children, a maximum of 12 months of market exclusivity period will be provided.

Draft for Comments mentioned the market exclusivity period for the first time in China. This represents that China will encourage the orphan drug development of rare diseases. However, combined with the European and American countries market exclusivity system regulations and implementation experience, this paper puts forward the following thoughts and suggestions. First, it is necessary to clarify the applicable scope of market monopoly of rare disease drugs in light of the national conditions of rare disease drugs in China, so as to improve the accessibility of rare disease drugs as soon as possible. Second, it should be clear whether the exclusive period of the children’s drug market can operate in tandem with the exclusive period of the rare disease drug market. Third, define the concept and definition criteria of the key elements of market exclusivity, like the definition of “same breed,” the “same indication” and “clinical advantage.”

Recently, the National Health Commission of China have postulated a series of positive policies to encourage the drug development of rare diseases, including the Set Standards of National Medical Center for Rare Diseases released on 27 December 2022, the goal of rare disease quality control improvement in 2023 released in February 2023, and the List of National Medical Quality Control Center for Rare Diseases and Members of its Expert Committee released in April 2023. These policies focus on the setting standards of national rare disease medical center and the quality control of rare diseases, which provides a guarantee for improving the efficiency of rare disease diagnosis and treatment.

With the continuous improvement of relevant policies, China will continue to encourage the development and innovation of drugs for rare diseases, including rare tumors, and find a development path for the treatment of rare diseases that is in line with China’s national conditions. Overall, these favorable regulatory and in-market policies indicate that the spring of drug development on rare diseases in China is coming.
