# Supplementary material for: Seven-year exclusivity and beyond for drugs of rare diseases in China
Source: Front Pharmacol. 2023 Oct 12;14:1223056. doi: 10.3389/fphar.2023.1223056 (PMC10600485; doi:10.3389/fphar.2023.1223056)
Supplement: Supplementary file 1 [file Table1.DOCX]

TableS1. 22 rare disease drugs included in the 2019-2021 National Health Insurance catalog of China

| Drug Name | Indication | Approved time |
| --- | --- | --- |
| Selexipag | Idiopathic Pulmonary Hypertension | 2019 |
| Riociguat | Idiopathic Pulmonary Hypertension | 2019 |
| Macitentan | Idiopathic Pulmonary Hypertension | 2019 |
| Bosentan | Idiopathic Pulmonary Hypertension | 2019 |
| Teriflunomide | Multiple Sclerosis | 2019 |
| Miglustat | Niemann-Pick Disease | 2019 |
| Filgrastim | Severe Congenital Neutropenia | 2019 |
| Levocarnitine | Primary Carnitine Deficiency | 2019 |
| Entacapone | Parkinson's Disease (Young Type, Early Onset) | 2019 |
| Siponimod | Multiple Sclerosis | 2020 |
| Deutetrabenazine | Huntington's Chorea | 2020 |
| Fingolimode | Multiple Sclerosis | 2020 |
| Nintedanib | Idiopathic Pulmonary Fibrosis | 2020 |
| EdaravoneInjection | Amyotrophic Lateral Sclerosis | 2020 |
| Ambrisentan | Idiopathic Pulmonary Hypertension | 2020 |
| Fampridine | Multiple Sclerosis | 2021 |
| Firazyr | Hereditary Angioedema | 2021 |
| Human coagulation factor factor Ⅹ | Haemophilia | 2021 |
| Synonyms | Idiopathic Cardiomyopathy | 2021 |
| Agalsidase Alfa | Fabry Disease | 2021 |
| Nusinersen | Spinal Muscular Atrophy | 2021 |
| Ebolocumab | Familial Hypereholesterolemia | 2021 |
